# The taxonomy and diversity of *Platerodrilus* (Coleoptera, Lycidae) inferred from molecular data and morphology of adults and larvae

**DOI:** 10.3897/zookeys.426.7398

**Published:** 2014-07-17

**Authors:** Michal Masek, Ladislav Bocak

**Affiliations:** 1 Department of Zoology, Faculty of Science, Palacky University, 17. listopadu 50, 771 46 Olomouc, Czech Republic

**Keywords:** Oriental Region, net-winged beetles, morphology, molecular phylogeny, taxonomy

## Abstract

The Oriental neotenic net-winged beetles attracted attention of biologists due to conspicuous large-bodied females; nevertheless phylogenetic relationships remain contentious and only a few species are known in both the fully metamorphosed males and neotenic females. The phylogenetic analyses and morphology of larvae and adults provide data for investigation of relationships and species delineation. *Platrilus* Kazantsev, 2009, *Platerodriloplesius* Wittmer, 1944, and *Falsocalochromus* Pic, 1942 are synonymized to *Platerodrilus* Pic, 1921. *Platrilus hirtus* (Wittmer, 1938) and *Pl. crassicornis* (Pic, 1923) are transferred to *Platerodrilus* Pic, 1921. *Platerodrilus hoiseni* Wong, 1996 is proposed as a junior subjective synonym of *Falsocalochromus ruficollis* Pic, 1942. *Platerodrilus* is divided in three species-groups: *P. paradoxus*, *P. major*, and *P. sinuatus* groups defined based on the shape of genitalia and molecular phylogeny. The following species are described: *Platerodrilus foliaceus* **sp. n.**, *P. wongi* **sp. n.** (*P. paradoxus* group); *P. ngi* **sp. n.**, *P. wittmeri* (*P. major* group), *P. ijenensis* **sp. n.**, *P. luteus* **sp. n.**, *P. maninjauensis* **sp. n.**, *P. montanus* **sp. n.**, *P. palawanensis* **sp. n.**, *P. ranauensis* **sp. n.**, *P. sibayakensis* **sp. n.**, *P. sinabungensis* **sp. n.**, *P. talamauensis* **sp. n.**, and *P. tujuhensis* **sp. n.** (*P. sinuatus* group). *P. korinchiana robinsoni* Blair, 1928 is elevated to the species rank as *P. robinsoni* Blair, 1928, **stat. n.** The conspecific semaphoronts are identified using molecular phylogeny for *P. foliaceus* **sp. n.**, *P. tujuhensis* **sp. n.**, *P. montanus* **sp. n.**, *P. maninjauensis* **sp. n.**; additional female larvae are assigned to the species-groups. Diagnostic characters are illustrated and keys are provided for *P. paradoxus* and *P. major* groups.

## Introduction

The platerodriline net-winged beetles are one of elateroid lineages with modified female morphology (Wong 1996, Bocak et al. 2008, Masek et al. 2014). The adult males are fully metamorphosed (Figs 4–17). In contrast, females do not pupate and the sexually mature females remain larviform ("trilobite larvae", Figs 2–3, 32–43, Mjöberg 1925, Wong 1996, Bocak and Matsuda 2003). The taxonomic situation is complicated by the fact that *Platerodrilus* males and females have been observed in copula only twice (Mjöberg 1925, Wong 1996).

The taxonomy of the neotenic lineages has quite short history despite the fact that the trilobite larvae were first time described in the 19^th^ century (Perty 1831, Candèze 1861). *Platerodrilus* Pic, 1921 was proposed for five species and although compared with *Plateros* Bourgeois, 1879 (Lycidae), the new taxon was placed in the distantly related Drilidae (now Drilini in Elateridae; Kundrata and Bocak 2011). Mjöberg (1925) erected *Duliticola*, described both sexes of *Duliticola paradoxa* Mjöberg, 1925 and discussed the possibility to establish Duliticolinae in Lycidae. Only recently the trilobite larvae attracted further students. Wong (1996) reported another case of a male and a female observed in copula and studied most M. Pic's types deposited in the Paris Museum, but his work remained unpublished (Wong 1998). Kazantsev (2002) described the subfamily Duliticolinae, but the name is unavailable and replaced by Miniduliticolinae (Kazantsev 2005). Kazantsev (2002) designated the type species of *Platerodrilus*, considered *Duliticola* as its junior synonym and later described a new genus *Platrilus* Kazantsev, 2009 which corresponds to *Platerodrilus* sensu Wong (1998). The subgenus *Platerodriloplesius* Wittmer, 1941 was elevated to the genus rank by Kazantsev (2002). These taxa are based on flabellate antennae (*Platerodriloplesius*) or the unique shape of male genitalia (*Platrilus*) and their relationships have remained contentious.

The DNA data represent an independent source of information for species delineation (Vuataz et al. 2011) and for identification of the conspecific semaphoronts (Ahrens et al. 2007). We present the molecular phylogeny of *Platerodrilus* and compare the results with morphology of adults and larvae to solve taxonomy of *Platerodrilus*. As a result, we describe new species and discuss their relationships.

## Methods

### Morphological taxonomy

Adult males and female larvae were used for morphological descriptions. A part of specimens used for morphological study was sequenced and labelled with the GenBank voucher numbers in the format UPOL + six-letter/number code and the status of all type specimens were designated with red labels (ICZN 1999). The codes are listed in examined material (Table 1). A. T. C. Wong studied many species in mid 1990's, but the types have not yet been returned to the Paris museum. Therefore, we redescribe only species, which are currently available in the types or are described as new. Other species were redescribed by Wong (1998) and Kazantsev (2009). All morphological measurements were taken using the ocular grid of an Olympus SZX-16 binocular microscope.

**Table 1.** Taxonomic coverage, locality data and GenBank accession numbers.

Species	Voucher UPOL+	Local. data	*rrnL*
**Outgroup**			
*Benibotarus nigripennis*	000572	Japan	DQ181001
*Benibotarus spinicoxis*	000573	Japan	DQ181002
*Dictyoptera elegans*	000570	Japan	DQ181375
*Dictyoptera speciosa*	000571	Japan	DQ181000
*Libnetis granicollis*	001012	Japan	DQ181033
*Libnetis* sp.	001002	Sumatra	DQ181030
*Libnetis* sp.	001008	Malaysia	DQ181031
*Libnetis* sp.	000L02	Sabah	DQ180964
*Lycoprogenthes* sp.	000801	Sumatra	DQ181021
*Lycoprogenthes* sp.	000802	Java	DQ181022
*Lycoprogenthes* sp.	000805	Sumatra	DQ181023
*Lycoprogenthes* sp.	000358	Java	DQ180996
*Pyropterus nigroruber*	000574	Japan	DQ181003
*Lyropaeus* sp.	VP0016	India	KC736885
*Lyropaeus* sp.	VP0017	India	KC736886
*Lyropaeus* sp.	VP2312	India	KC736887
*Lyropaeus dominator*	VP0003	Malaysia	KC736882
*Lyropaeus optabilis*	VP0004	Malaysia	KC736883
*Lyropaeus optabilis*	000585	Malaysia	DQ181014
*Lyropaeus ritsemae*	VP0001	Sumatra	KC736880
*Lyropaeus ritsemae*	VP0006	Sumatra	KC736884
*Lyropaeus rubrostriatus*	000L11	Malaysia	DQ180968
*Lyropaeus waterhousei*	VP0002	Sumatra	KC736881
*Lyropaeus waterhousei*	000584	Sumatra	DQ181013
**Ingroup**			
*Horakiella emasensis*	001043	Malaysia	DQ181036
*Macrolibnetis depressus*	VP0050	Malaysia	KF802467
*Macrolibnetis depressus*	000L21	Malaysia	DQ180976
*Pendola* sp.	000M45	Java	DQ180984
*Platerodrilini* gen. sp.	VP0009	Malaysia	KF802457
*Platerodrilini* gen. sp.	VP0010	Sumatra	KF802480
*Platerodrilini* gen. sp.	VP0012	Malaysia	KF802458
*Platerodrilini* gen. sp.	VP0030	India	KF802462
*Platerodrilini* gen. sp.	VP0031	India	KF802463
*Platerodrilini* gen. sp.	VP0034	India	KF802464
*Platerodrilus curtus*	001380	Mindanao	KF625997
*Platerodrilus curtus*	001381	Mindanao	KF626073
*Platerodrilus curtus*	001383	Mindanao	KF626074
*Platerodrilus curtus*	VP0014	Mindanao	KF802459
*Platerodrilus curtus*	VP2316	Mindanao	KF802479
*Platerodrilus angustatus*	001388	Sumatra	KF626001
*Platerodrilus corporaali*	001373	Sumatra	KF625991
*Platerodrilus foliaceus*	000588	Borneo	DQ181017
*Platerodrilus foliaceus*	000589	Borneo	EF143214
*Platerodrilus ijenensis*	000586	Java	DQ181015
*Platerodrilus luteus*	001379	Sumatra	KF625996
*Platerodrilus major*	001387	Sumatra	KF626000
*Platerodrilus maninjauensis*	001374	Sumatra	KF625992
*Platerodrilus maninjauensis*	001377	Sumatra	KF625994
*Platerodrilus maninjauensis*	001386	Sumatra	KF626075
*Platerodrilus maninjauensis*	VP2303	Sumatra	KF802470
*Platerodrilus maninjauensis*	VP2306	Sumatra	KF802473
*Platerodrilus montanus*	001371	Sumatra	KF625989
*Platerodrilus montanus*	VP2308	Sumatra	KF802475
*Platerodrilus ngi*	VP0021	Singapore	KF802461
*Platerodrilus ranauensis*	000587	Sumatra	DQ181016
*Platerodrilus robinsoni*	001378	Sumatra	KF625995
*Platerodrilus sibayakensis*	001372	Sumatra	KF625990
*Platerodrilus sibayakensis*	001389	Sumatra	KF802552
*Platerodrilus* sp.	000L01	Sabah	DQ180963
*Platerodrilus* sp.	VP0044	Sabah	KF802465
*Platerodrilus* sp.	VP2301	Sabah	KF802468
*Platerodrilus* sp.	VP0020	Malaysia	KF802460
*Platerodrilus* sp.	VP0047	Sumatra	KF802466
*Platerodrilus* sp.	VP2302	Malaysia	KF802469
*Platerodrilus* sp.	VP2304	Thailand	KF802471
*Platerodrilus* sp.	VP2307	Sumatra	KF802474
*Platerodrilus* sp.	VP2309	Malaysia	KF802476
*Platerodrilus* sp.	VP2310	Malaysia	KF802477
*Platerodrilus* sp.	VP2311	Laos	KF802478
*Platerodrilus* sp.	MB1382	Palawan	EF625998
*Platerodrilus talamauensis*	001375	Sumatra	KF626072
*Platerodrilus talamauensis*	001376	Sumatra	KF625993
*Platerodrilus tujuhensis*	001385	Sumatra	KF625999
*Platerodrilus tujuhensis*	VP2305	Sumatra	KF802472

**Abbreviations and depositories.** Descriptions: BL–length of body; WH–width at humeri; PL–length of pronotum; PW–width of pronotum; Ediam–maximum eye diameter; Edist–minimum interocular distance in frontal part of cranium. Depositories: LMBC–Voucher collection of the Laboratory of Molecular Systematics, Faculty of Science UP, Olomouc; BMNH–Natural History Museum, London; MNHP–Museum d'histoire naturelle, Paris; ZRCS–Zoological Reference Collection, Raffles Museum of Biodiversity Research, NUS; KMTC–Kiyoshi Matsuda Collection, Takarazuka.

### Laboratory methods and phylogenetic analyses

Total DNA was extracted using Wizard SV96 kit (Promega Inc.) and primers 16a (5'-CGCCTGTTTAACAAAAACAT-3'), 16b (5'-CCGGTCTGAACTCAGATCATGT-3') and ND1A (5'-GGTCCCTTACGAATTTGAATATATCCT-3') were used for PCR amplification of the 530–810 base pairs of *rrnL*, which showed the best results in identification of immature stages (Levkanicova and Bocak 2009). The setting of PCR reaction were described by Sklenarova et al. (2013). Purified PCR products were sequenced by an ABI 3130 automated sequencer using the Big Dye Terminator Cycle Sequencing Kit 1.1.

### Sequence handling and phylogenetic analyses

Sequences were edited using the Sequencher 4.8 software package (Gene Codes Corp.). The *rrnL* mtDNA fragment was aligned using ClustalW 1.83 (Thompson et al. 1994), BlastAlign 1.2 (Belshaw and Katzourakis 2005) under default parameters, and Muscle 3.6 (Edgar 2004) under the gap opening parameter 2600 and gap extension parameter 240. The phylogenetic analyses were carried out under the maximum likelihood criterion using the RAxML 7.2.3 (Stamatakis et al. 2005) and the bootstrap support of branches (BS) assessed by analyzing 100 pseudoreplicates. All genes and codon positions in the protein coding fragments were partitioned. The model was proposed by jModelTest 2.1.2 (Posada 2008). The dataset was additionally analyzed using MrBayes 3.2.2 (Huelsenbeck 2000). The MCMC was set for independent variability of parameters in individual coding and non-coding genes under the GTR+I+G model. Two runs, each with four chains ran simultaneously for 40×10^6^ generations, with trees being sampled every 1000^th^ generation, all fragments were partitioned and unlinked. The first 6–9×10^6^ trees were discarded as burn-in and posterior probabilities (PP) at nodes were determined from the remaining trees.

**Figure 1.**
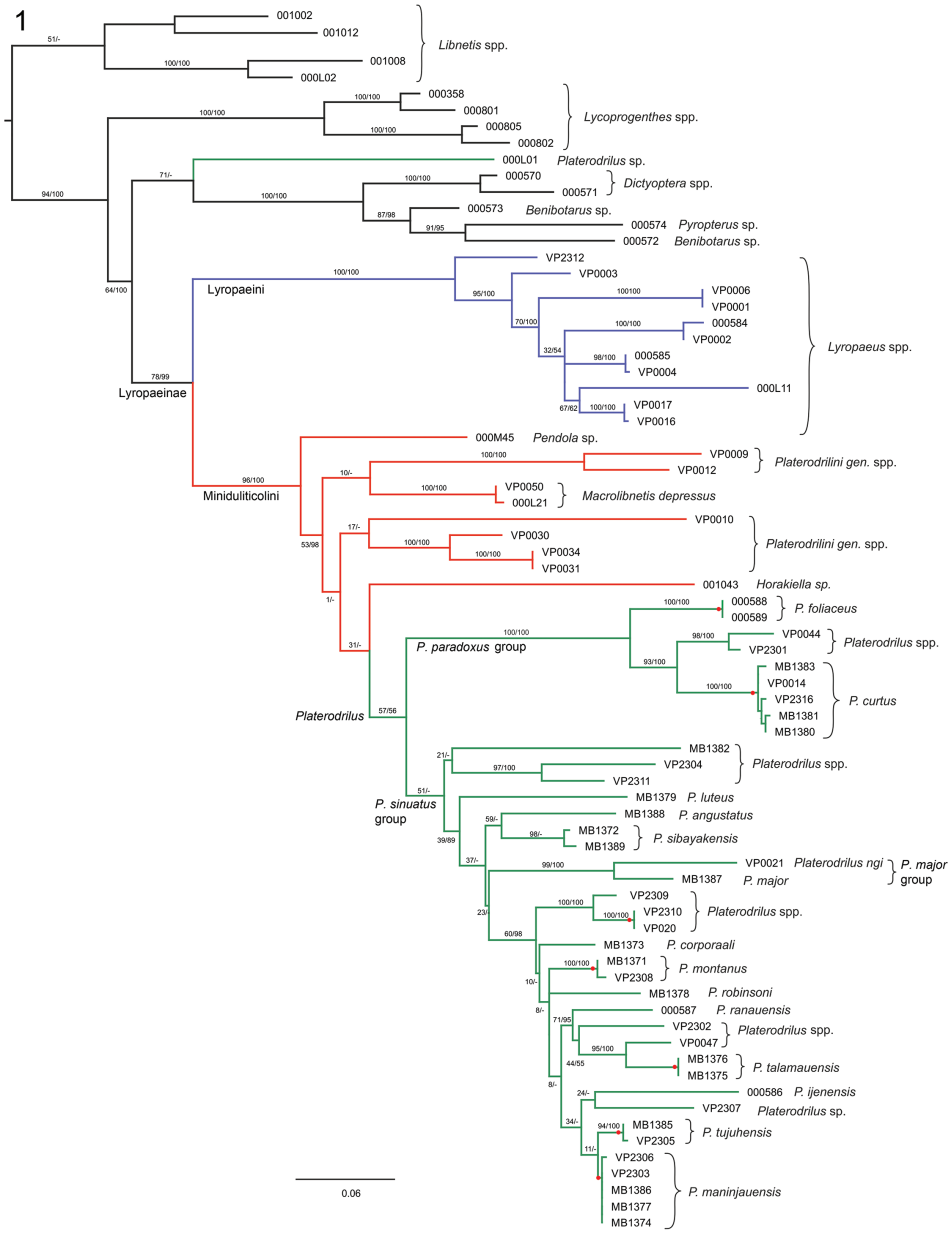
Phylogenetic hypothesis for *Platerodrilus* Pic, 1921 based on a maximum likelihood analysis of the Muscle alignment. Numbers at the branches are maximum likelihood bootstrap values and Bayesian posterior probabilities. The red dots designate GMYC species clusters.

The ultrametric tree was produced from the tree depicted in Fig. 1 using r8s software (Sanderson 2002) and the GMYC method as implemented in SPLITS (http://www.rforge.r-project.org/projects/splits/) was applied to the ultrametric tree.

## Results

### Sequence variation and phylogeny

The DNA sequences of *rrnL* were produced for 73 specimens. The dataset of aligned *rrnL* sequences contained 530–723 homologous positions depending on the applied alignment procedure; 253–267 characters were parsimony informative. The topologies produced from BlastAlign, Muscle and Clustal alignments analyzed under maximum likelihood method and Bayesian inference identified the same strongly supported principal clades of Lyropaeinae and although topologies differed somewhat with respect to the deeper nodes of Miniduliticolini, all analyses recovered *Pendola*, *Macrolibnetis*, *Horakiella* and related taxa as deeper splits of Miniduliticolini and *Platerodrilus* as a terminal lineage (Fig. 1). One terminal, *Platerodrilus* sp. 000L01 was recovered outside Lyropaeinae and we consider its position as an artefact of the single marker analysis. The species was found in relationships to another *Platerodrilus* in the six-gene analysis of Lycidae (Bocak et al. 2008). Therefore this taxon is not considered in the further discussion.

The *Platerodrilus paradoxus* and *Platerodrilus major* clades were well supported in all analyses except by analysis of the Clustal alignment (BS 100%, PP 52–100% and BS 97–99%, PP 52–100%, respectively). The *Platerodrilus sinuatus* group (including the nested *Platerodrilus major* group) obtained much lower support (BS 46–56%, PP < 89%). The species level clusters and relationships of closely related species were regularly well supported (Fig. 1).

The GMYC analysis of the normalized tree was used as an independent test for morphology based delineation of species. The analysis suggested the clusters designated as *Platerodrilus tujuhensis* and *Platerodrilus maninjauensis* (uncorrected genetic distance 1.4%) as separate species and these are well supported also by morphological differences (see Taxonomy). Similarly, two separate species were inferred for two specimens of *Platerodrilus sibayakensis* (uncorrected genetic distance 0.8%) and these do not differ in any morphological character and were collected in the same region.

### DNA identification of immature stages

The origin of large-bodied neotenic larvae was recovered in three unrelated lineages: *Lyropaeus*, *Macrolibnetis depressus* + unidentified species from India and *Platerodrilus* (Fig. 1). Males and female larvae of four species, i.e. *Platerodrilus foliaceus*, *Platerodrilus maninjauensis*, *Platerodrilus montanus* and *Platerodrilus tujuhensis* clustered with conspecific males in clades with very high bootstrap support. Additionally, a number of larvae was assigned to the species groups in relationships to the previously described species (Fig. 1). We found that the species of the *Platerodrilus paradoxus* clade share pronotum without glabrous prominent tubercles (Figs 1, 34, 40). Similarly, the species of the *Platerodrilus sinuatus* group from continental Asia (Fig. 1) have smooth terga (VP2304, VP2311). The glabrous tubercles in the discs of the thoracic terga are present only in the lineage of Sumatran and Malay species of the *Platerodrilus sinuatus* group (Fig. 1; terminals VP2308, VP2302, VP2307, VP0047 etc.). The robust, vermiform larva (Fig. 43) clustered with species of the *Platerodrilus major* clade (Fig. 1).

## Discussion

### Supergeneric classification of *Platerodrilus* and related genera.

The results confirm that Lyropaeini (i.e., *Lyropaeus* Waterhouse, 1878 *sensu lato*) is an independent lineage with the large-bodied neotenic females and *Platerodrilus* belong to a sister-clade of Lyropaeini along with *Macrolibnetis*, *Horakiella*, and *Pendola* (Fig. 1). Most of these genera are also known only from males but no large bodied female has been assigned to them and their females are probably larviform but similar in body size to males. The only miniduliticoline taxon with the large-bodied female except *Platerodrilus* is *Macrolibnetis* (Levkanicova and Bocak 2009).

The subfamiliar and tribal classification of *Platerodrilus* and related genera has been ambiguous. Mjöberg (1925) used the name Duliticolinae, but stated that the formal description should be postponed. Therefore, Duliticolinae Mjöberg, 1925 is an unavailable name. Kazantsev (2002) erected subfamily Duliticolinae without any description and with type genus *Platerodrilus* as a single genus classified in the new tribe, when he considered *Duliticola* as a junior synonym. According to the articles 13.1 and Articles 11.7.1.1 and 64 (ICZN 1999) the name Duliticolinae Kazantsev, 2002 is unavailable. Kazantsev (2002) further proposed the name Miniduliticolini for *Miniduliticola* Kazantsev 2002, but the description is uninformative: "The hypothesized apomorphies of the genus *Miniduliticola* gen. n., particularly glabrous elytra with no trace of longitudinal costae or tubercles support the erection of a new tribe". The single type specimen of *Miniduliticola* is damaged and there is no information available on male genitalia. The name Miniduliticolini Kazantsev, 2002 became the oldest available name for a clade which is recovered as a sister clade to Lyropaeini Bocak and Bocakova, 1989 (Fig. 1).

Kazantsev (2005) proposed Platerodrilini to replace Duliticolini Kazantsev, 2002. In this case, he provided description of the new taxon: "The Platerodrilini tr. n. is tentatively included in Miniduliticolinae. The hypothesized apomorphy of the new tribe distinguishing it from Miniduliticolini is the reticulated elytra." (Kazantsev 2005). Therefore, we have two available names for supergeneric taxa, one based on a single damaged specimen bearing characters correlated with small body (i.e. simplified structures, Bocak et al. 2014) and the second name without any delineation, based on *Platerodrilus*, which represents a crown branch in the current molecular phylogeny. As the position of Miniduliticolini was considered tentative in Duliticolinae sensu Kazantsev (2002) and also Platerodrilini were tentatively placed in Miniduliticolini (Kazantsev 2002), the names are not connected to any phylogenetic hypothesis to define their limits and relationships of *Miniduliticola* remain unknown. Therefore, we propose to use Miniduliticolini for designation of the whole clade in a sister position to Lyropaeini including *Platerodrilus*, *Pendola*, *Horakiella* and *Macrolibnetis* (Fig. 1). If Miniduliticolini belong to any other lineage, the sister-group to Lyropaeini would be designated as Platerodrilini.

### Generic delineation of *Platerodrilus* and related genera.

**Figures 2–3.**
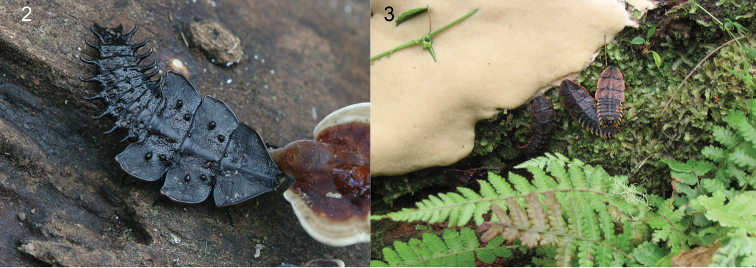
**2** Female larvae of *Platerodrilus*. **3** *Platerodrilus* sp. from Gn. Sinabung, Sumatra, ditto from Gn. Apo, Mindanao.

*Platerodrilus* Pic, 1921, *Duliticola* Mjöberg, 1925, *Macrolibnetis* Pic, 1938, *Platerodriloplesius* Wittmer, 1941, *Falsocalochromus* Pic, 1942 and *Platrilus* Kazantsev, 2009 are available genus-group names referring to the platerodriline net-winged beetles with large-bodied neotenic females occurring in South East Asia (Fig. 2). The current results confirm that *Macrolibnetis* Pic, 1938 represents a distant lineage and does not belong to the *Platerodrilus* clade [considered as a synonym of *Platerodrilus* by Bocakova (2001) and Kazantsev (2002), reinstated by Bocak and Bocakova (2008)].

The other Miniduliticolini with large-bodied neotenic females form a clade designated as *Platerodrilus* in Fig. 1. The deepest split of *Platerodrilus* consists of a clade of *Platerodrilus curtus*, *Platerodrilus foliaceus* and several unidentified larvae (Figs 37–38). Their male genitalia (Figs 44–47) resemble those of *Platerodrilus paradoxus* (see Kazantsev 2009) and the larvae do not have glabrous tubercles in the disc of the pronotum. These characters place *Platerodrilus paradoxus* (type species of *Duliticola*) in the clade designated as *Platerodrilus paradoxus* group in Fig. 1. Further, based on morphology, two species *Platerodrilus svetae* and *Platerodrilus wongi* (Fig. 5) are placed here. This group is a monophyletic lineage in Fig. 1, but we do not reinstate the name *Duliticola* Mjöberg, 1925 as the present sampling is limited and the genus cannot be recognized using external characters.

**Figures 4–17.**
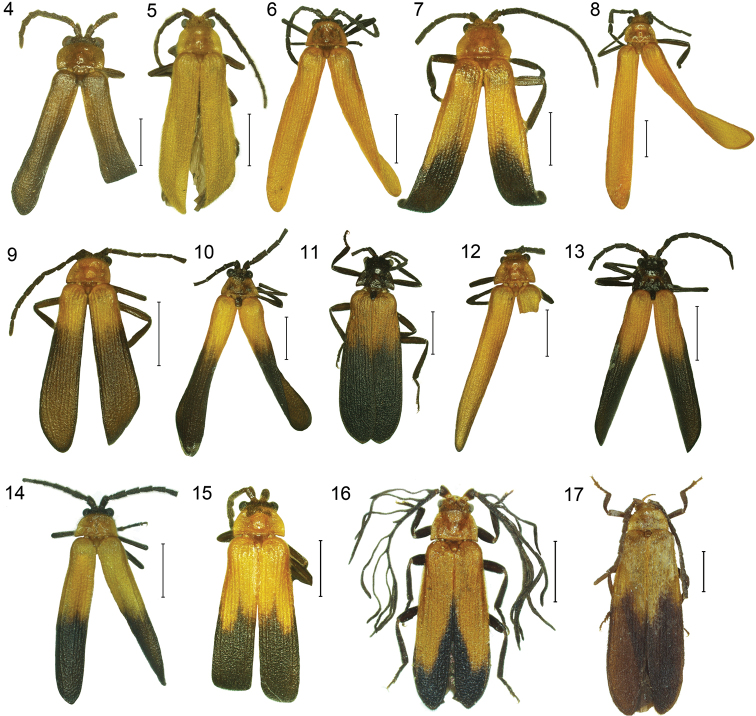
Adult male, general appearance: **4** *Platerodrilus foliaceus* **5** *Platerodrilus wongi* **6** *Platerodrilus robinsoni* **7** *Platerodrilus maninjauensis* **8** *Platerodrilus luteus* **9** *Platerodrilus ranauensis* **10** *Platerodrilus sibayakensis* **11** *Platerodrilus sinabungensis* **12** *Platerodrilus tujuhensis* **13** *Platerodrilus montanus* **14** *Platerodrilus ijenensis* **15** *Platerodrilus talamauensis* **16** *Platerodrilus palawanensis* **17** *Platerodrilus wittmeri*. Scales 2 mm.

The sister clade to the *Platerodrilus paradoxus* clade contains *Platerodrilus* species with two types of male genitalia: (a) the phallus with short, densely pubescent parameres (Figs 48–51) and (b) the phallus slender, curved, parameres with long membranous apical process (Figs 52–72). We designate these groups as *Platerodrilus major* and *Platerodrilus sinuatus* groups (Fig. 1). The *Platerodrilus major* group (Figs 1, 17, 30, 48–51) contains species placed in *Platrilus* by Kazantsev (2009). This lineage represents a crown clade within *Platerodrilus sinuatus* group (Fig. 1). This assemblage was designated as *Platerodrilus* sensu Wong (1998), when *Platerodrilus major* Pic, 1921 was proposed as a type genus of *Platerodrilus* and *Duliticola paradoxa* Mjöberg, 1925 for *Duliticola* to keep both names valid (invalid designations in the unpublished manuscript by Wong 1998). Kazantsev (2002) designated *Platerodrilus sinuatus* Pic, 1921 as a type species of *Platerodrilus* and considered as *Duliticola* a junior synonym of *Platerodrilus*. Subsequently, he erected a separate genus *Platrilus* Kazantsev, 2009. As a subordinate lineage, *Platrilus* cannot be accepted in classification and is proposed to be a junior synonym of *Platerodrilus*.

The *Platerodrilus sinuatus* group contains species from continental Asia, which form a deep split (terminals VP2304, VP2311) and further a group of species from the Sundaland and Palawan (Fig. 1). Larvae from continental Asia do not have any tubercles in the thoracic terga, similarly to the *Platerodrilus paradoxus* and *Platerodrilus major* group, only the species from Sumatra, Java and Malay Peninsula have the glabrous tubercles both in the disc and posterior margins of thoracic terga (Figs 32–33, 39). The males of these species can be assigned to continental or Sundaland lineages only with DNA data. Therefore, we propose to group them in the *Platerodrilus sinuatus* group despite paraphyly of the assemblage.

**Figures 18–34.**
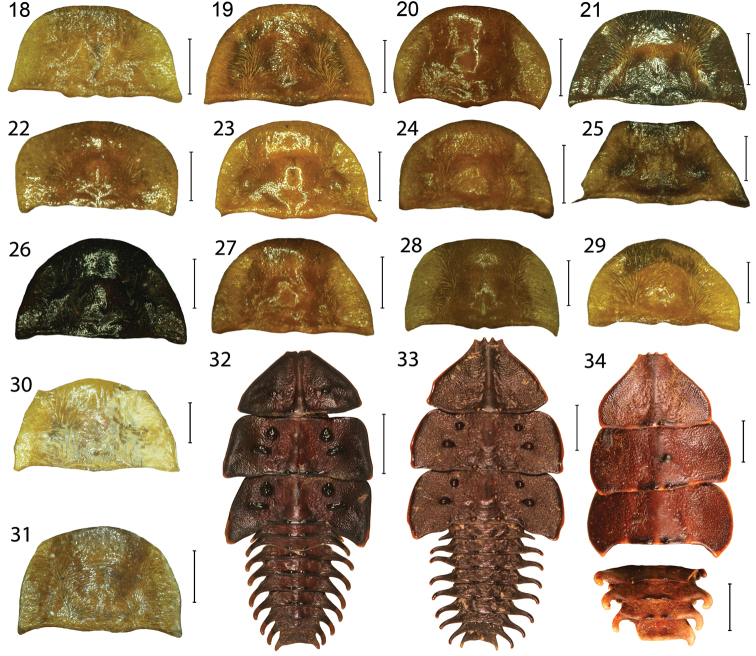
Male pronotum of *Platerodrilus*. **18** *Platerodrilus ijenensis* **19** *Platerodrilus robinsoni* **20** *Platerodrilus maninjauensis* **21** *Platerodrilus montanus* **22** *Platerodrilus foliaceus* **23** *Platerodrilus luteus* **24** *Platerodrilus ranauensis* **25** *Platerodrilus sibayakensis* **26** *Platerodrilus sinabungensis* **27** *Platerodrilus tujuhensis* **28** *Platerodrilus wongi* **29** *Platerodrilus talamauensis* **30** *Platerodrilus wittmeri* **31** *Platerodrilus palawanensis*. Larva, general appearance: **32** *Platerodrilus maninjauensis* **33** *Platerodrilus montanus* **34** *Platerodrilus paradoxus*. Scales 0.5 mm (Figs **18–31**); Scales 5 mm (**32–34**).

**Figures 35–43.**
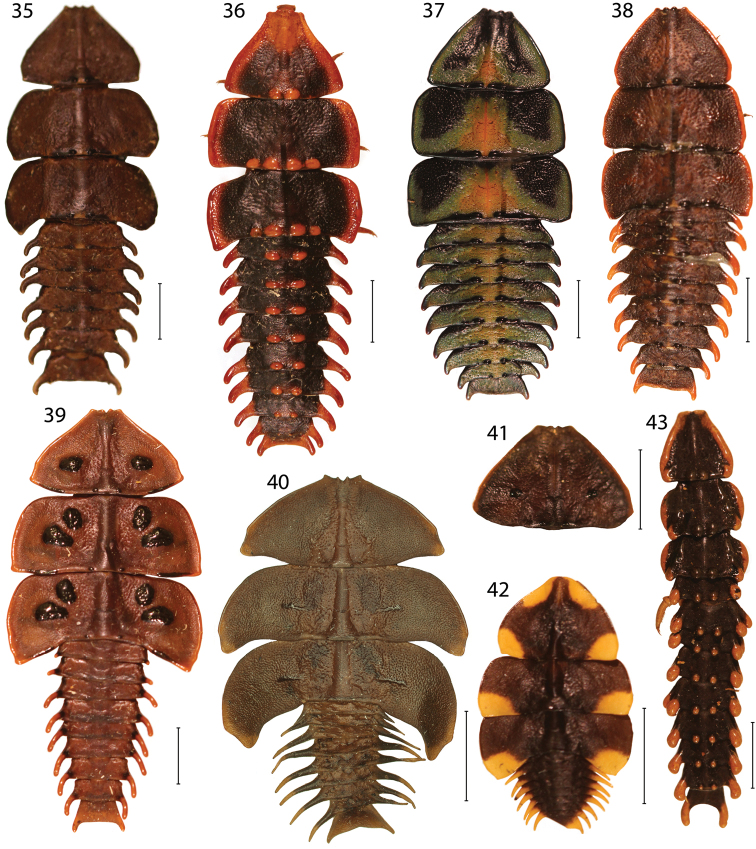
Larvae of *Platerodrilus* and *Macrolibnetis*: **35–38** *Platerodrilus* spp. **39** *Platerodrilus ruficollis* **40** *Platerodrilus foliaceus* **41** *Platerodrilus tujuhensis* **42** *Macrolibnetis depressus* **43** *Platerodrilus ngi*. Scales 5 mm.

Further two genus-group names were proposed for species now placed in *Platerodrilus*. *Platerodriloplesius* was erected for taxa with flabellate male antennae (Wittmer 1944). The male genitalia of *Platerodrilus bicolor* (Wittmer, 1941) (type species of *Platerodriloplesius*) resemble those of *Platerodrilus paradoxus*. In contrast, genitalia of *Platerodrilus palawanensis* sp. n. (Figs 69–70) and *Platerodrilus borneensis* (Wittmer, 1966) (both species having the flabellate antennae) indicate their relationships to *Platerodrilus sinuatus* group (Figs 52–72). Morphology of genitalia indicate that the species classified in *Platerodriloplesius* belong to different clades and *Platerodriloplesius* in Kazantsev's sense is a polyphyletic typological assemblage based on a highly variable morphology of male antennae, which might be used for pheromone communication, and therefore their surface is expanded by lamellae to house a higher number of olfactory sensors. As the type species of *Platerodriloplesius* belongs to the *Platerodrilus paradoxus* group, *Platerodriloplesius* is a junior synonym of *Duliticola* and *Platerodrilus*.

**Figures 44–55.**
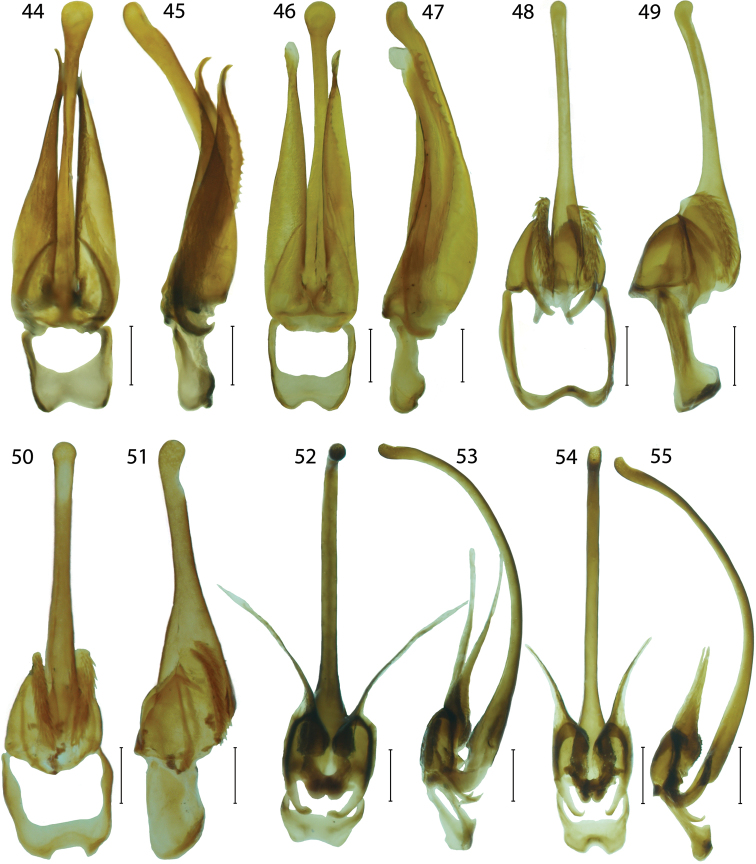
Male genitalia of *Platerodrilus*: **44–45** *Platerodrilus wongi* **46–47** *Platerodrilus foliaceus* **48–49** *Platerodrilus major* **50–51** *Platerodrilus wittmeri* **52–53** *Platerodrilus talamauensis* **54–55** *Platerodrilus ranauensis*. Scales 0.25 mm.

**Figures 56–66.**
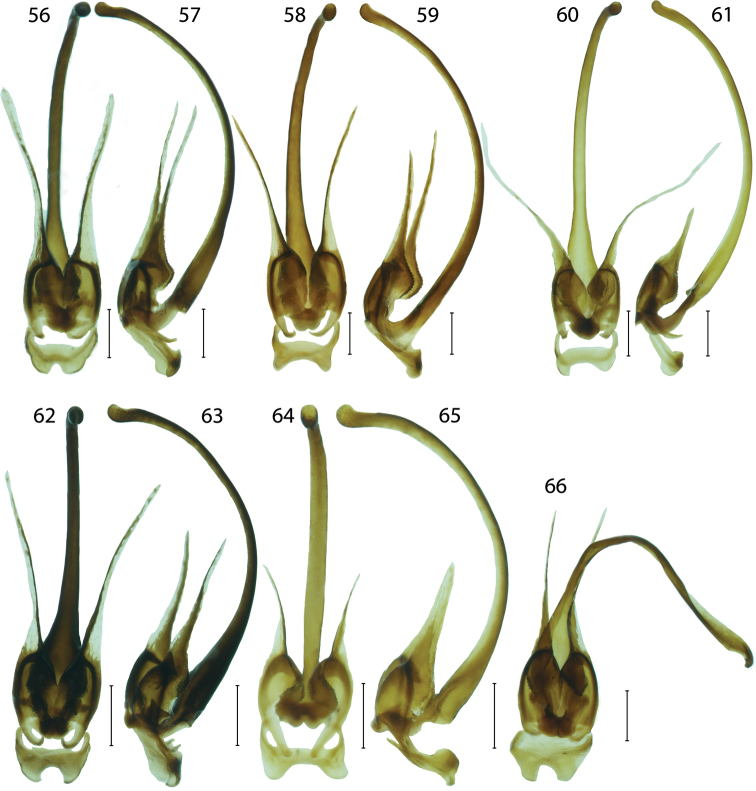
Male genitalia of *Platerodrilus*: **56–57** *Platerodrilus tujuhensis* **58–59** *Platerodrilus sinabungensis* **60–61** *Platerodrilus maninjauensis* **62–63** *Platerodrilus robinsoni* **64–65** *Platerodrilus ijenensis* **66** *Platerodrilus montanus*. Scales 0.25 mm.

**Figures 67–72.**
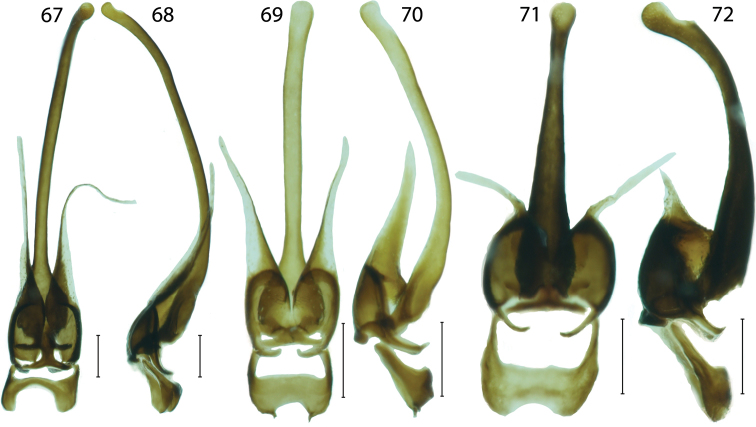
Male genitalia of *Platerodrilus*: **67–68** *Platerodrilus luteus* **69–70** *Platerodrilus palawanensis* **71–72** *Platerodrilus sibayakensis*. Scales 0.25 mm.

Pic (1942) described *Falsocalochromus* in the supposed relationships to *Calochromus* Guérin-Méneville, 1833. The described species *Falsocalochromus ruficollis* Pic, 1942 is conspecific with *Duliticola hoiseni* Wong, 1996 from the *Platerodrilus sinuatus* group and the *Falsocalochromus* is a junior synonym of *Platerodrilus*.

To sum up, we propose to consider *Duliticola*, *Platerodriloplesius*, *Platrilus* and *Falsocalochromus* to be junior synonyms of *Platerodrilus*. *Platerodriloplesius* represents a polyphyletic assemblage and *Platrilus* is a terminal branch supported by unique apomorphies rendering *Platerodrilus* in a paraphylum. As these taxa cannot be assigned to species groups without dissection of male genitalia, the proposed generic classification results in a definition of an easily recognisable monophyletic assemblage.

Rapid morphological divergence in male genitalia is widespread and results largely from sexual selection (Eberhard 2010). Therefore, delineations based on highly divergent genital morphology can lead to proposal of genus-rank taxa when the group of species sharing divergent genitalia represents only a terminal subclade. The molecular phylogeny revealed such pattern in broadly defined *Platerodrilus*, where three types of male genitalia are encountered. On the other hand, we can see low divergence in genitalia within *Platerodrilus* subclades, e.g., the *Platerodrilus sinuatus* group. The diverging populations of *Platerodrilus* are in allopatry and the reinforcement of the reproductive barriers cannot take place. Probably as a result, the male genitalia are similar within species groups consisting of allopatrically distributed species (Wong 1998).

## Taxonomy

### Platerodrilus

Taxon classificationAnimaliaColeopteraLycidae

Pic, 1921

Platerodrilus Pic, 1921: 13.
Platerodrilus sinuatus Pic, 1921 (subsequent designation by Kazantsev 2002: 6). Type species.
Duliticola Mjöberg, 1925: 133; Kazantsev 2002: 6.
Duliticola paradoxa Mjöberg, 1925 (by monotypy). Type species.
Platerodriloplesius Wittmer, 1941: 196 (as a subgenus of *Platerodrilus* Pic, 1921); Kazantsev 2009 (genus rank); syn. n.
Platerodriloplesius bicolor Wittmer, 1966 (by monotypy). Type species.
Falsocalochromus Pic, 1942: 3, syn. n.
Falsocalochromus ruficollis Pic, 1942: 4 (by monotypy). Type species.
Platrilus Kazantsev, 2009: 61, syn. n.
Platerodrilus hirtus Wittmer, 1938 (by original designation). Type species.

#### Adult male.

**Diagnosis.** *Platerodrilus* differs from most Miniduliticolini in the stout body (6–11 mm) and characteristic types of male genitalia (Figs 44–72). The morphologically similar *Pendola* has genitalia resembling those of *Lyropaeus* (Bocak 2002). *Lyropaeus* differs in 10-segmented antennae and absent transverse costae on elytra.

**Description.** Male. Body 5.8–10.8 mm, flat, slightly widened posteriorly, densely pubescent. Pronotum and elytra bicoloured, uniformly yellow or black (Figs 4–17).

Head small, prognathous to slightly hypognathous, partly retracted in pronotum. Eyes hemispherically prominent, frontal interocular distance longer than maximum eye diameter. Labrum sclerotized, transverse, separated from clypeus, mandibles slender, long, slightly curved, incisor margin simple, without teeth, maxilla tiny, with setose mala, stipes plate-like, cardo vestigial, palpifer short, maxillary palpi 4-segmented, palpomere 1 shortest, about twice longer than palpifer, palpomere 2 longest, slender, palpomere 3 slightly longer than wide, apical palpomere slender, drop-like, with slender apical part. Labium reduced, mentum plate-like, formed by single sclerite, ligula absent, palpi 3-segmented, basal palpomeres subequal, rectangular to slightly longer than wide, apical palpomere twice longer than wide at base, pointed to apex.

Antennal tubercles present, usually strongly prominent. Antennae 11-segmented, slightly surpassing middle of elytral length, dark coloured, never with apical antennomeres pale, antennae usually weakly serrate, a few species with flabellate antennae. Scapus pear-like, robust, pedicel and antennomere 3 subequal in length, antennomeres of serrate antennae flattened, from antennomere 4 gradually slenderer, apical antennomere long, parallel-sided. Flabellate antennae with lamellae of antennomeres 3–10 longer than body of antennomere; whole antennae with dense erected pubescence.

Pronotum transverse with prominent to obtuse anterior angles (Figs 18–31), without carinae. Lateral margins elevated, straight to widely rounded, frontal angles sometimes inconspicuous, posterior angles mostly sharp, posterior margin bisinuate. Pronotum with deep depression along lateral margins, with sparse, long, erected pubescence. Scutellum longer than wide, triangular, simply rounded at apex. Elytra flat, slightly widened posteriorly, with well marked humeri; elytral costae inconspicuous, only costa 2 and 4 traceable in whole elytra, costa 4 forming humeral edge, other costae apparent at humeri, undefined in rest of elytral length. Two rows of inconspicuous, irregular cells traceable between costae, giving appearance of secondary costa in some parts of elytra. Elytra with dense, long pubescence. Wings fully developed. Legs slender, coxae long, movable, trochanters very slender, femora flat, robust, tibiae slenderer than femora, tarsi slender, 5-segmented, tarsomeres 3–4 with small pads, tarsomere 5 long, slender, claws simple. Male abdomen slender, shorter and narrower than elytra, 8 segmented, tergum 8 simply rounded at apex, sternum 8 with strengthened lateral margins at base and membranous window basally. Male genitalia trilobate, variable in relative length of phallus and parameres, phallus stout, almost straight and sometimes laterally compressed or phallus long, very slender, sickle like (Figs 52–72); parameres reaching to half or four fifths of phallic length, with fine spines along internal margin, fully sclerotized (Figs 44–47) or considerably shorter, apically with membranous process, phallobase short, emarginate basally (Figs 48–51).

#### Female mature larva.

**Diagnosis.** Body shape characteristic ("trilobite larva" Figs 32–43). Two body types are present: the flat, wide (Figs 32–42) and robust, vermiform (Fig. 43). Although different in general appearance these larvae share common diagnostic characters: the fossa antennalis closed, separated from the mouth-parts by pleurostoma (the pleurostoma absent from other lycids); slender, longitudinal sclerite present ventrally of pleurostoma; mala sclerotized. The apical antennomere with several peg-like processes. The complex, oval meso- and metathoracic spiracles are cribriform, the sieve plate with multiple openings. They are situated in large depressions. Similar larvae of *Lyropaeus* differ in the shape of the apical antennomere and both *Macrolibnetis* and *Lyropaeus* do not have spiracular cavities in abdominal segments (Masek et al. 2014).

**Description.** Body wide, considerably flattened due to extensively projected lateral plates (Figs 32–41) or slender with postero-lateral processes (Fig. 43), usually dark brown, cryptically coloured, some species aposematically coloured with brightly coloured patches. Head prolonged, rounded anteriorly; epicranium consists of dorsal and pleural plates, membranous between plates. Complete fossa antennalis dorsally limited by epicranium, ventrally by sclerotized pleurostoma. Longitudinal sclerite situated ventrally of pleurostoma. Basal antennomere very short, apical antennomere with several peg-like processes ventrally and more extensive, sclerotized area dorsally. Mala sclerotized, with peg-like process. Cervical membrane extensive, with pigmented patches postero-ventrally. Pronotum trapezoid, terga with considerably widened lateral plates and sometimes with tubercles at posterior margin. Prosternum prolonged, episterna extensive, attached to prosternum. Extensive spiracular plates with spiracular openings at margin and bottom of extensive cavity in both, meso- and metathorax. Legs slender, relatively long, trochanters divided in two parts. Abdomen with large lateral processes, spiracular openings on margin and bottom of cavities in segments A1–A8. Sterna A1–A8 with slender postero-lateral processes, upper pleurites extensive, with similar process at outer posterior angle. Lower pleurites very small, with short process only in segments A3–A8. Segment A9 widest at apex, with short, fixed urogomphi (Figs 35–40, 43).

#### Remark.

Sexually mature larviform females observed only by Mjöberg (1925) and Wong (1996) were not available to us. The collected larvae did not pass the final ecdysis and their maturity is supposed on the basis of their body length. Only several lower instar larvae were collected and they differ from later instars in shorter and partly missing processes and absence of spiracular cavities.

#### Biology.

The information on biology was given by Wong (1996) and Bocak and Matsuda (2003).

### List of species

#### Species group *Platerodrilus paradoxus* Mjöberg, 1925

**Diagnosis.** The species group *Platerodrilus paradoxus* was recovered as a monophyletic assemblage representing one of principal *Platerodrilus* lineages. The species of this group share male genitalia with long, slender and completely sclerotized parameres (Figs 44–47). Known females do not have any glabrous tubercles in the middle of thoracic terga (Figs 34–38, 40), one species from Mt. Kinabalu has tubercles only at posterior margins of thoracic terga (Fig. 40). The following species are classified here: *Platerodrilus bicolor* Wittmer, 1941, *Platerodrilus curtus* Pic, 1931, *Platerodrilus foliaceus* sp. n., *Platerodrilus paradoxus* Mjöberg, 1925, *Platerodrilus piceicollis* Pic, 1943, *Platerodrilus strbai* Kazantsev, 2009, *Platerodrilus svetae* Kazantsev, 2009, *Platerodrilus wongi* sp. n.

**Distribution.** Most species are known from Borneo and the Philippines, only *Platerodrilus wongi* sp. n. occurs in Sumatra.

**Remark.** As only *Platerodrilus curtus*, *Platerodrilus foliaceus* and several unidentified female larvae were available for DNA isolation, the monophyly of this lineage needs further support before validity of the name *Duliticola* Mjöberg, 1925 can be reconsidered.

##### Platerodrilus paradoxus

Taxon classificationAnimaliaColeopteraLycidae

(Mjöberg, 1925)

Duliticola paradoxa Mjöberg, 1925: 134.
Platerodrilus paradoxus : Kazantsev 2002: 6.

###### Material examined.

Syntype. Male (BMNH), Borneo. Syntype. Female (BMNH), Lundu, Sarawak, G. E. Bryant, 6. 1. 14.

###### Diagnosis.

*Platerodrilus paradoxus* belongs to a group of Bornean species with robust and long parameres. The male of *Platerodrilus paradoxus* resembles *Platerodrilus foliaceus* but differs in the slender apex of parameres. Additionally these species differ in larval morphology (Figs 34, 40).

###### Redescription.

Male. Body 7 mm, dark brown, only humeri and elytral suture slightly lighter. Head small, with hemispherically prominent eyes, head with eyes wider than frontal margin of pronotum, eye diameter 1.9 times frontal interocular distance. Antennae compressed, covered with long, erected, dense pubescence. Pronotum flat, without carinae, 1.8 times wider than long at midline, frontal margin widely rounded anteriorly, frontal angles obtuse, lateral margins almost straight, posterior margin bisinuate. Elytra flat, parallel-sided, elytral costae inconspicuous, elytra 2.6 times longer than width at humeri. Legs slender, compressed, densely pubescent. Male genitalia with robust parameres, phallus slightly curved with bulbous tip. Parameres stout with hooked tip, apical half of ventral edge serrate. Phallobase wide, deeply emarginate.

Female larva. Body flat and wide (Fig. 34), pronotum parallel-sided at base, then gradually tapering to front, triangular, without any glabrous tubercles in disc, only small tubercles in middle part of posterior margins of thoracic segments, in middle of anterior pronotal margin four subequal tubercles; mesothorax strongly transverse, with rounded lateral margins and straight posterior margin, posterior angles obtuse, metathorax similar in shape with more acutely projected posterior angles. Abdomen with short, robust lateral processes.

###### Measurements.

Male. BL 6.9 mm, PL 1.0 mm, PW 1.9 mm, HW 2.0 mm, Edist 0.85 mm, Ediam 0.44 mm. Larva. BL 53.0 mm, PL 8.7 mm, PW 13.4 mm.

###### Distribution.

Malaysia: Sarawak. Known only from the type locality.

##### Platerodrilus foliaceus sp. n.

Taxon classificationAnimaliaColeopteraLycidae

http://zoobank.org/53AA2216-605A-4A64-A10B-C58AEF0B8E31

Figs 4 , 22 , 40 , 46–47

###### Material examined.

Holotype. Male (LMBC, UPOL 000589), Borneo, Central Kalimantan Prov., 60 km SE Muara Teweh, 1°20'25"S, 115°20'16"E, 24.–28. Jun. 2001, 150 m. Paratypes. 12 females, same locality data (LMBC, UPOL 000588).

###### Diagnosis.

*Platerodrilus foliaceus* belongs to the *Platerodrilus paradoxus* group and the male resembles *Platerodrilus paradoxus* in general appearance. *Platerodrilus foliaceus* differs in the rounded apex of parameres (Figs 46–47). The female larvae of *Platerodrilus foliaceus* are very flat and have much slenderer lateral processes of abdominal segments than *Platerodrilus paradoxus* (Figs 34, 40).

###### Description.

Male. Body small-sized, brown, head, antennae, legs except bases of femora and apical three fifths of elytra dark brown (Fig. 4). Head small, with hemispherically prominent eyes, head with eyes slightly wider than frontal margin of pronotum, eye diameter 1.7 times frontal interocular distance. Antennae compressed, covered with long, erected, dense pubescence, length of antennomere 3 0.7 times antennomere 2. Pronotum flat, without carinae, 1.7 times wider than long at midline, frontal margin slightly projected anteriorly, frontal angles obtuse, but apparent, lateral margins rounded, posterior margin slightly bisinuate (Fig. 22). Elytra flat, parallel-sided, elytral costae inconspicuous, elytra 2.9 times longer than width at humeri. Legs slender, compressed, densely pubescent. Male genitalia with robust parameres, phallus slightly curved with bulbous tip. Parameres stout with hooked tip, apical half of ventral edge serrate. Phallobase wide, deeply emarginate (Figs 46–47).

Female larva. Body extremely flat and wide (Fig. 40), pronotum triangular, without any glabrous tubercles, in middle of anterior margin four subequal tubercles; mesothorax strongly transverse, with rounded lateral margins and moderately projected posterior angles, metathorax similar in shape with more acutely projected posterior margins. Abdomen with very slender and long lateral processes.

###### Measurements.

Male. BL 6.0 mm, PL 0.9 mm, PW 1.5 mm, HW 1.7 mm, Edist 0.78 mm, Ediam 0.46 mm. Larva. BL 19.3 mm, PL 5.1 mm, PW 11.2 mm.

###### Distribution.

Indonesia: Kalimantan. Known only from the type locality.

###### Etymology.

The specific epithet refers to the flat body shape of the female larva.

###### Remark.

The males and female larvae were identified as conspecific on the basis of highly similar sequences of *rrnL* (Fig. 1).

##### Platerodrilus wongi sp. n.

Taxon classificationAnimaliaColeopteraLycidae

http://zoobank.org/67F26E3D-C5E3-4E35-A630-B6A636DB969D

Figs 5 , 28 , 44–45

###### Material examined.

Holotype. Male (LMBC), Sumatra Utara, Brastagi, Gn. Sibayak, 19–23. Feb. 1998, 700–2000 m.

###### Diagnosis.

*Platerodrilus wongi* is a single species of the *Platerodrilus paradoxus* group occurring in Sumatra. It resembles *Platerodrilus curtus* from the Philippines in the uniformly yellow elytra, but has relatively shorter parameres (Figs 44–45).

###### Description.

Body brown, head, pronotum, mesoscutellum and elytra yellow (Fig. 5). Head small, with eyes is slightly wider than frontal margin of pronotum, antennal tubercles deeply separated. Eyes hemispherically prominent, frontal interocular distance 2.4 times eye diameter. Antennae slender, compressed, reaching two thirds of elytra length, antennomere 3 1.1 times antennomere 2. Head and antennae covered with short dense pubescence. Pronotum transverse, 1.6 wider than length at midline. Anterior margin only slightly projected, anterior angles well marked, lateral margins almost straight, posterior margin shallowly bisinuate (Fig. 28). Elytra parallel-sided, with inconspicuous carinae. Elytra 3.0 times longer than width at humeri, elytra widest in apical sixth. Legs compressed, densely pubescent. Male genitalia with laterally compressed, slightly curved phallus with bulbous tip, parameres stout, long, with hooked tip, apical half of ventral edge serrated, phallobase wide, deeply emarginate (Figs 44–45).

###### Measurements.

BL 7.4 mm, PL 1.0 mm, PW 1.6 mm, HW 1.9 mm, Edist 0.91 mm, Ediam 0.38 mm.

###### Distribution.

Indonesia: Northern Sumatra.

###### Etymology.

The specific epithet is a patronym in honour of Alvin T. C. Wong.

##### The key to identification of males from the *Platerodrilus paradoxus* species group

**Table:** 

1	Parameres short, reaching slightly over half of phallic length	*Platerodrilus strbai* Kazantsev
–	Parameres reaching almost to the apex of the phallus	2
2	Male antennae flabellate	*Platerodrilus bicolor* Wittmer
–	Male antennae serrate	3
3	Whole elytra yellow (Fig. 5)	4
–	Elytra dark brown or light brown with dark coloured apical part	5
4	Parameres reaching to five sixths of the phallic length	*Platerodrilus curtus* Pic
–	Parameres reaching to three fourths of the phallic length	*Platerodrilus wongi* sp. n.
5	Only apical part of elytra dark coloured, pronotum black	*Platerodrilus piceicollis* Pic and *Platerodrilus svetae* Kazantsev*
–	Whole elytra dark brown, at most the narrow humeral part slightly lighter brown	6
6	Body slender, inner margin of parameres serrate only in its apical half	*Platerodrilus foliaceus* sp. n.
–	Body robust, inner margin of parameres serrate in three quarter of their length	*Platerodrilus paradoxus* (Mjöberg)

* We failed to find any distinguishing character between *Platerodrilus svetae* and *Platerodrilus piceicollis*. The type of *Platerodrilus svetae* is deposited in a private collection and unavailable for study.

#### Species group *Platerodrilus major* Pic, 1921

**Diagnosis.** The species group *Platerodrilus major* is a monophyletic assemblage representing a terminal branch, which includes the species placed in *Platrilus* Kazantsev, 2009 and it is represented in the current analysis by *Platerodrilus major* and *Platerodrilus ngi* (Fig. 1). The group is characterized by the short and setose parameres without long membranous apical processes (Figs 48–51). The following species are placed here: *Platerodrilus atronotatus* Pic, 1943, *Platerodrilus crassicornis* Pic, 1923, *Platerodrilus hirtus* Wittmer, 1938, *Platerodrilus major* Pic, 1921, *Platerodrilus ngi* sp. n. and *Platerodrilus wittmeri* sp. n. The species described by M. Pic were redescribed by Wong (1998).

##### Platerodrilus ngi sp. n.

Taxon classificationAnimaliaColeopteraLycidae

http://zoobank.org/638DD7A0-68EC-499D-A6BA-16560A6D2808

Fig. 43

###### Material examined.

Holotype. Male (LMBC), Singapore, Bukit Timah and Central Water Catchment, 19.–22. May 2013, 50–100 m, E. Jendek and O. Šauša leg. Paratypes. Female larvae, 6 spec., Malaysia, Pahang, Tioman, Kg. Tekek–Juara trail, 50–300 m, 2°49'10"N, 104°10'21"E, 29. Mar.–2. Apr. 2013, same locality data, 4.–16. Mar. 1998, L. Dembicky and P. Pacholatko (LMBC); 1 spec., Singapore, Sime Road swamp, 30. Oct. 2008 (ZRCS);1 spec., Singapore, Bukit Timah Nature Reserve, A. T. C. Wong 1993 (ZRCS); 1 spec., Singapore, Sime Road, C. Lee (ZRCS, #6.20969, 1993.7277, 1993.7278).

###### Diagnosis.

*Platerodrilus ngi* is the only representative of the *Platerodrilus major* group known from Singapore and Tioman. It differs in dark red colouration of the pronotum and humeral two thirds of elytra from *Platerodrilus atronotatus* from the Malay Peninsula. *Platerodrilus atronotatus* has the black pronotum. Additionally, the phallus of *Platerodrilus atronotatus* is slender and antennomeres 3 and 4 short and much wider.

###### Description.

Male. Body small, dark brown to black, head, prothorax, mesoscutellum and basal two thirds of elytra dark red; whole body with dense, short, pubescence. Head small, including eyes slightly wider than frontal margin of pronotum. Eyes hemispherically prominent, frontal interocular distance 1.8 times eye diameter. Antennae robust at base, compressed, reaching two thirds of elytral length, antennomere 3 0.6 times antennomere 2, antennomere 3 as long as wide at apex. Pronotum flat, 1.7 times wider than long at midline. Anterior margin almost straight, anterior angles sharply marked, posterior margin bisinuate. Elytra slightly wider posteriorly, elytra 2.8 times longer than width at humeri; only slightly widened posteriorly, elytral costae conspicuous. Legs compressed, densely pubescent. Male genitalia with straight phallus and setose parameres, short phallobase slightly shorter than parameres.

Female larva. Body slender, parallel-sided (Fig. 43), pronotum triangular, with two basal, dark coloured, glabrous tubercles; mesothorax slightly transverse, without projected posterior angles, metathorax similar in shape. Abdomen with robust, short lateral processes and fixed urogomphi.

###### Measurements.

Male. BL 6.9 mm, PL 0.9 mm, PW 1.6 mm, HW 2.1 mm, Edist 0.86 mm, Ediam 0.48 mm. Larva. BL 30.2 mm, PL 5.7 mm, PW 5.6 mm.

###### Distribution.

Singapore, Malaysia: Pahang. Biology and female specimens collected in Singapore were reported by Lok (2008).

###### Etymology.

The species name is a patronym in honour of Peter Ng.

##### Platerodrilus wittmeri sp. n.

Taxon classificationAnimaliaColeopteraLycidae

http://zoobank.org/F646300B-C3B4-48BA-98B2-9BB9571C762B

Figs 17 , 30 , 50–51

###### Material examined.

Holotype. Male (LMBC), Java, K. O. Blawan, Ijen Plateau, Jul. 1940, 900–1500 m, H. Lucht coll.

###### Diagnosis.

*Platerodrilus wittmeri* is the only bicoloured species from the *Platerodrilus major* group in Java. It resembles *Platerodrilus major* from Northern Sumatra in colouration but differs in the shorter phallobase (Figs 48–49, 50–51).

###### Description.

Male. Body medium-sized, dark brown, head, prothorax, mesoscutellum and basal half of elytra testaceous; antennae, legs, apical half of elytra dark brown to black (Fig. 17). Head small, including eyes slightly wider than frontal margin of pronotum. Eyes hemispherically prominent, frontal interocular distance 1.9 times eye diameter. Antennae slender, compressed, reaching two thirds of elytral length, antennomere 3 0.7 times antennomere 2. Head and antennae with dense, short, pubescence. Pronotum flat, 1.9 times wider than long at midline. Anterior margin slightly projected anteriorly, anterior angles sharply marked, prominent, posterior margin bisinuate (Fig. 30). Elytra almost parallel-sided, 3.5 times longer than width at humeri; only slightly widened posteriorly, elytral costae inconspicuous. Legs compressed, densely pubescent. Male genitalia with straight phallus and setose parameres, short phallobase slightly shorter than parameres (Figs 50–51).

###### Measurements.

BL 10.1 mm, PL 1.1 mm, PW 2.0 mm, HW 2.6 mm, Edist 0.95 mm, Ediam 0.49 mm.

###### Distribution.

Indonesia: Java.

###### Etymology.

The specific epithet is a patronym in honour of the late W. Wittmer, who donated the specimen to the senior author in 1992.

##### The key to identification of males from the *Platerodrilus major* species group

**Table:** 

1	Metathorax orange brown	*Platerodrilus crassicornis* Pic
–	Metathorax dark brown to black	2
2	Phallobase shorter than parameres (≤ 0.95 times length of parameres)	*Platerodrilus wittmeri* sp. n.
–	Phallobase longer than parameres (≥ 1.05 times length of parameres	3
3	Phallus short and stout, about 0.90 the combined length of parameres and phallobase, pronotum similar in colour to humeral part of elytra	4
–	Phallus long and slenderer, about as long as the combined length of parameres and phallobase, pronotum blac	*Platerodrilus atronotatus* Pic
4	Antennomere 4 wide, about as long as wide at apex	*Platerodrilus ngi* sp. n.
–	Antennomere 4 at least 1.25 times longer than wide at apex	5
5	The expanded base of phallus in lateral view mostly hidden by parameres, phallobase deeply emarginate basally, elytra black apically	*Platerodrilus major* Pic
–	The expanded base of phallus in lateral view exposed, considerably widened, phallobase shallowly emarginate basally, elytra testaceous	*Platerodrilus hirtus* Wittmer

#### Species group *Platerodrilus sinuatus* Pic, 1921

**Diagnosis.** The group *Platerodrilus sinuatus* is a paraphyletic assemblage consisting of *Platerodrilus luteus*, the Indo-Burmese species (represented in the analyzed dataset by female larvae VP2304, VP2311), *Platerodrilus palawanensis* from Palawan, *Platerodrilus indicus* from Assam and Nepal and the terminal lineage of *Platerodrilus* from Sundaland (Figs 1–2). All species have a long, slender, considerably curved phallus and short parameres with the membranous apical process (Figs 52–72). The following species are placed in this species group: *Platerodrilus angustatus* Pic, 1921, *Platerodrilus apicalis* Pic, 1936, *Platerodrilus atricolor* Pic, 1938, *Platerodrilus corporaali* Pic, 1921, *Platerodrilus grootaerti* Kazantsev, 2009, *Platerodrilus holynskae* Kazantsev, 2009, *Platerodrilus inapicalis* Pic, 1937, *Platerodrilus indicus* Wittmer, 1966, *Platerodrilus luteus* sp. n., *Platerodrilus maninjauensis* sp. n., *Platerodrilus montanus* sp. n., *Platerodrilus palawanensis* sp. n., *Platerodrilus ranauensis* sp. n., *Platerodrilus reductus* Pic, 1926, *Platerodrilus rotundicollis* Wittmer, 1938, *Platerodrilus ruficollis* Pic, 1942, *Platerodrilus rufus* Pic, 1924, *Platerodrilus sinuatus* Pic, 1921, *Platerodrilus talamauensis* sp. n., *Platerodrilus tujuhensis* sp. n., *Platerodrilus sibayakensis* sp. n. and *Platerodrilus sinabungensis* sp. n. The female larvae of species occurring in continental Asia north of the Isthmus of Kra have terga without glabrous tubercles similarly to the species of the *Platerodrilus paradoxus* clade. The group of species from the Malay Peninsula, Sumatra and Java are characterized by larvae with glabrous tubercles in thoracic terga (Figs 1, 32–33, 39, 41). The male genitalia are similar in the shape of the basal part of the phallus and phallobase. The minute differences are difficult to describe in a form of the identification key and the DNA data were used for confirmation of the species delineation in several cases.

##### Platerodrilus ijenensis sp. n.

Taxon classificationAnimaliaColeopteraLycidae

http://zoobank.org/21013FB1-3B9C-42F2-879D-A1CAC5AF4EED

Figs 14 , 18 , 64–65

###### Material examined.

Holotype. Male (LMBC, 000586), Java, Ijen N. P., 12 km W of Sodora, 3–5. May 2001, 1000 m.

###### Diagnosis.

*Platerodrilus ijenensis* is the only Javanese species of *Platerodrilus sinuatus* group with bicoloured elytra. It resembles in colour pattern the Sumatran species *Platerodrilus corporaali*, which differs in the reddish coloured head and basal antennomeres, and the very short antennomere 3.

###### Description.

Body and head dark brown to black, pronotum, mesoscutellum and elytra in humeral half orange, apical part of elytra dark brown to black (Fig. 14). Head small, including eyes narrower than pronotum at posterior angles, antennal tubercles slightly prominent. Eyes hemispherically prominent, eye diameter 1.8 times frontal interocular distance. Antennae slender, compressed, length of antennomere 3 1.2 times antennomere 2. Pronotum transverse, 1.8 wider than long at midline, anterior margin slightly projected forward, anterior angles marked, lateral margins almost straight, posterior margin bisinuate (Fig. 18). Elytra 3.3 times longer than width at humeri, elytra parallel-sided, elytral costae inconspicuous. Legs slender, compressed, densely pubescent. Male genitalia with slender, curved phallus and small rounded parameres bearing slender membranous process, phallobase wide, narrowly and deeply emarginate (Figs 64–65).

###### Measurements.

BL 7.2 mm, PL 0.9 mm, PW 1.6 mm, HW 1.8 mm Edist 0.70 mm, Ediam 0.40 mm.

###### Distribution.

Indonesia: Java.

###### Etymology.

The specific name refers to the type locality.

##### Platerodrilus korinchianus

Taxon classificationAnimaliaColeopteraLycidae

(Blair, 1928)

Duliticola korinchiana Blair, 1928: 181.
Platerodrilus korinchianus (Blair, 1928): Kazantsev 2009.

###### Material examined.

Lectotype (hereby designated). Male (BMNH), Sumatra, Korinchi, 4500 ft, N. 1914, K. G. Blair. Paralectotype. Female (BMNH), same locality data.

###### Diagnosis.

The male genitalia are missing. Therefore, only information on external morphology can be compared. The orange pronotum and humeral part of elytra resemble *Platerodrilus corporaali* or *Platerodrilus maninjauensis*, but no similar species is known from the Kerinci massif.

###### Redescription.

*Male.* Body black, head dark brown, pronotum and basal quarter of elytra orange; antennae, and legs dark brown to black. Head small, antennal tubercles weak, eyes hemispherically prominent, frontal interocular distance 2.3 times maximum eye diameter. Antennae slender, compressed, densely pubescent, antennomere 3 as long as antennomere 2. Pronotum transverse, 1.5 times wider than long, anterior margin almost straight, frontal angles conspicuous, lateral margins almost straight, posterior angles acute, surface mat at margins, slightly glabrous in middle. Elytra parallel-sided, elytral costae weak, covered with dense pubescence. Male genitalia missing.

###### Measurements.

PL 1.3 mm, PW 1.9 mm, HW 2.6 mm, Edist 0.84 mm, Ediam 0.36 mm.

###### Distribution.

Indonesia: Sumatra, Jambi, Kerinci massif.

###### Remark.

The lectotype is damaged (the apical half of elytra and abdomen are missing). *Platerodrilus korinchianus* differs in the shape of pronotum and colouration of elytra from *Platerodrilus tujuhensis* and *Platerodrilus robinsoni*. The paralectotype, a female larva, is very similar to the female larva of *Platerodrilus tujuhensis* from the same locality and might not be conspecific with the male specimen. As larvae are generally difficult to identify without DNA data, we prefer to designate a lectotype to keep status and preserve the validity of the name. The species can be misidentified as high diversity of neotenic net-winged beetles in the region was documented (Malohlava and Bocak 2010) and further species of *Platerodrilus* can occur in this locality.

##### Platerodrilus luteus sp. n.

Taxon classificationAnimaliaColeopteraLycidae

http://zoobank.org/6BE29EC6-3F39-4775-83EC-7BAB16828345

Figs 8 , 23 , 67–68

###### Material examined.

Holotype. Male (LMBC, UPOL 001379), Sumatra, Jambi Kersik Tua, Gn. Kerinci, 19.–22. Jan. 2005, 1600–2200 m.

###### Diagnosis.

*Platerodrilus luteus* resembles in general appearance the syntopically occurring *Platerodrilus robinsoni*, but these species are distantly related according to the recovered molecular phylogeny (Fig. 1). The male of *Platerodrilus luteus* differs from similarly coloured Sumatran *Platerodrilus* in the very wide and broadly emarginate phallobase (Fig. 67).

###### Description.

Body black, head dark brown, pronotum and elytra orange; antennae, and legs dark brown to black (Fig. 8). Head small, antennal tubercles weak, eyes hemispherically prominent, frontal interocular distance 2.8 times maximum eye diameter. Antennae slender, compressed, densely pubescent, antennomere 3 1.5 times longer than antennomere 2. Pronotum transverse, 1.7 times wider than long, frontal angles inconspicuous, lateral margins slightly convex, posterior angles acute (Fig. 23). Elytra parallel-sided, 3.7 times longer than width at humeri, elytral costae weak, covered with dense pubescence. Male genitalia with curved phallus; parameres short, rounded, with slender membranous processes; phallobase wide, deeply emarginate (Fig. 67–68).

###### Measurements.

BL 10.1 mm, PL 1.1 mm, PW 1.8 mm, HW 2.3 mm, Edist 0.83 mm, Ediam 0.45 mm.

###### Distribution.

Indonesia: Sumatra.

###### Etymology.

The specific epithet refers to yellow colouration of the body.

##### Platerodrilus maninjauensis sp. n.

Taxon classificationAnimaliaColeopteraLycidae

http://zoobank.org/6F62649B-E974-4BF7-BD30-BF13FD3A8681

Figs 7 , 20 , 32 , 60–61

###### Material examined.

Holotype. Male (LMBC, UPOL 001386), Sumatra, Barat Lake Maninjau, E coast, 12.–23. Jan. 2005, 800 m. Paratype. Male (LMBC, UPOL 001374), Sumatra, Barat Lake Maninjau, E coast, 12.–23. Jan. 2005, 800 m. Paratypes. Male, 2 females (LMBC, UPOL 001377), Sumatra, Barat, Pasaman, Gn. Talamau, 14.–15. Jan. 2005, 1000 m; female larva (LMBC, UPOL VP2303), Sumatra, Barat Lake Maninjau, E coast, 12.–23. Jan. 2005, 800 m.

###### Diagnosis.

*Platerodrilus maninjauensis* is a sister species to *Platerodrilus tujuhensis* from Northern Sumatra. These species differ in the colouration (Figs 7, 12) and the shape of the phallus and phallobase (Figs 56–57, 60–61). The female larvae of both species are similar and differ only in the relative size of the mesonotal tubercles, which are smaller in *Platerodrilus tujuhensis* (Figs 32, 41).

###### Description.

Male. Body medium-sized, dark brown; head, prothorax, mesoscutellum and basal three fifths of elytra orange; antennae, legs, apical two fifths of elytra dark brown to black (Fig. 7). Head small, including eyes narrower than frontal margin of pronotum. Eyes hemispherically prominent, eye diameter 2.2 times frontal interocular distance. Antennae slender, compressed, reaching two thirds of elytral length, antennomere 3 0.9 times antennomere 2. Head and antennae with dense, short, pubescence. Pronotum flat, 1.1 times wider than long at midline. Anterior margin widely rounded, anterior angles inconspicuous, posterior margin bisinuate (Fig. 20). Elytra almost parallel-sided, 3.5 times longer than width at humeri; slightly widened posteriorly, widest at apical fourth. Elytral costae inconspicuous. Legs compressed, densely pubescent. Male genitalia with short rounded parameres bearing slender membranous process. Phallus curved, phallobase wide, widely emarginate (Figs 60–61).

Female larva. Body flat, wide (Fig. 32), pronotum triangular, with two glabrous rounded tubercles postero-laterally, another two tubercles in middle of anterior margin; mesothorax strongly transverse, with rounded lateral margins and weakly projected posterior angles, laterally with four tubercles, upper rounded, lower transverse, metathorax similar in shape with more acutely projected posterior margins. Abdominal segments with slender and long lateral processes.

###### Measurements.

BL 7.0 mm, PL 0.9 mm, PW 1.6 mm, HW 1.7 mm, Edist 0.84 mm, Ediam 0.38 mm. Larva. BL 24.1 mm, PL 5.4 mm, PW 10.3 mm.

###### Distribution.

Indonesia: Sumatra.

###### Etymology.

The specific epithet refers to the type locality of the holotype.

##### Platerodrilus montanus sp. n.

Taxon classificationAnimaliaColeopteraLycidae

http://zoobank.org/A434CA37-4D6B-4055-92F6-A16A1D3CA9B5

Figs 13 , 21 , 33 , 66

###### Material examined.

Holotype. Male (LMBC, UPOL 001371), Sumatra Utara, Brastagi, Gn. Sibayak, 26. Jan.–1. Feb. 2005, 1600–2200 m. Paratype. Female larva (LMBC, UPOL VP2308), Sumatra Utara, Brastagi, Gn. Sinabung, 29.–30. Jan. 2005, 1400–2000 m.

###### Diagnosis.

*Platerodrilus montanus* and *Platerodrilus sinabungensis* are the only Sumatran species with the dark coloured pronotum. These species differ in the shape of the phallobase, when *Platerodrilus montanus* has the narrowly emarginate base (Figs 58–59, 66). The larva of *Platerodrilus montanus* has pronotum without any glabrous tubercles in the disc (Fig. 33).

###### Description.

Body medium-sized, dark brown to black, only basal half of elytra orange and pronotum with irregular light coloured patches in disc (Fig. 13). Head small, including eyes slightly narrower than frontal margin of pronotum. Eyes hemispherically prominent, eye diameter 2.4 times frontal interocular distance. Antennae compressed, length of antennomere 3 1.1 times antennomere 2. Head and antennae with dense, short, pubescence. Pronotum transverse, 1.9 times wider than long at midline; anterior margin slightly projected, lateral margins almost straight, anterior angles weakly marked, posterior margin of pronotum slightly bisinuate (Fig. 21). Elytra almost parallel-sided, elytra 3.5 times longer than width at humeri; slightly widened posteriorly, widest at apical fourth, elytral costae inconspicuous. Legs compressed, with dense pubescence. Male genitalia with short rounded parameres bearing slender membranous processes. Phallus curved, phallobase wide, deeply emarginate (Fig. 66).

Female larva. Body flat, wide (Fig. 33), pronotum triangular, without glabrous tubercles except two tubercles in middle of posterior margin; mesothorax strongly transverse, with rounded lateral margins and weakly projected posterior angles, laterally with four tubercles, upper rounded, lower only slightly transverse, metathorax similar in shape with more acutely projected posterior margins. Abdominal segments with slender and long lateral processes.

###### Measurements.

BL 8.2 mm, PL 0.9 mm, PW 1.8 mm, HW 2.1 mm, Edist 0.94 mm, Ediam 0.39 mm. Larva. BL 32.3 mm, PL 7.7 mm, PW 13.0 mm.

###### Distribution.

Indonesia: Sumatra, North Sumatra Province, Gn. Sibayak.

###### Etymology.

The specific epithet is derived from the Latin adjective *montanus* (mountainous) referring to the habitat of the species.

##### Platerodrilus palawanensis sp. n.

Taxon classificationAnimaliaColeopteraLycidae

http://zoobank.org/F7FBBEE1-3910-4258-AEE4-E819747B834C

Figs 16 , 31 , 69–70

###### Material examined.

Holotype. Male (LMBC, UPOL 001382), Philippines, Palawan, Tanabak river, 150 m, 10°02'49"N, 118°58'31"E, 2.–5. Jan. 2007, Bolm lgt. Paratypes, 8 males (LMBC), same locality data, 22. Dec. 1991; 3 paratypes. Males (KMTC), Philippines, Palawan, Brooke's point, 8. Dec. 2002, leg. F. A. Dacasin. Paratype. Male (KMTC), Philippines, Palawan, Brooke's point, 18. May 2003, leg. F. A. Dacasin. Paratype. Male (KMTC), Philippines, Palawan, Brooke's point, 15. Jan. 2005, leg. F. A. Dacasin.

###### Diagnosis.

*Platerodrilus palawanensis* sp. n. resembles *Platerodrilus borneensis* in flabellate antennae and these species differ in colouration. *Platerodrilus borneensis* is dark brown and *Platerodrilus palawanensis* bicoloured (Fig. 16). Additionally *Platerodrilus palawanensis* has the very short pronotum and straight frontal pronotal margin (Fig. 31).

###### Description.

Body medium-sized, dark brown to black, only pronotum, mesoscutellum and humeral two thirds of elytra orange; apical third of elytra and two thirds of elytral suture dark brown to black. Head small, including eyes apparently narrower than frontal margin of pronotum, antennal tubercles robust, deeply separated. Eyes hemispherically prominent, eye diameter 1.9 times frontal interocular distance. Antennae flabellate, antennomere 3 with long process, pubescent, length of antennomere 3 1.6 times antennomere 2. Pronotum strongly transverse, 1.5 times wider than long at midline, anterior margin straight, with prominent anterior angles, lateral margins almost straight, posterior margin bisinuate, surface of disc mat, finely punctuate, with dense short pubescence. Elytra 2.9 times longer than width at humeri, elytra almost parallel-sided; slightly widened posteriorly, widest at apical fourth, elytral costae inconspicuous. Legs compressed, with dense pubescence. Male genitalia with curved phallus, short rounded parameres bearing slender membranous processes; phallobase wide, deeply emarginate (Figs 69–70).

###### Measurements.

BL 7.1 mm, PL 1.0 mm, PW 1.6 mm, HW 1.9 mm, Edist 0.66 mm, Ediam 0.34 mm.

###### Distribution.

Philippines: Palawan.

###### Etymology.

The specific epithet refers to the type locality of the holotype.

##### Platerodrilus ranauensis sp. n.

Taxon classificationAnimaliaColeopteraLycidae

http://zoobank.org/F09DE9AF-6800-4A06-AF7D-B82F3207217E

Figs 9 , 24 , 54–55

###### Material examined.

Holotype. Male (LMBC, UPOL 000587), Sumatra, SW coast of Ranau Lake, 1–4. Jun. 2001, 1200 m.

###### Diagnosis.

*Platerodrilus ranauensis* was found as a sister species to *Platerodrilus talamauensis* (Fig. 1) and these species differ in the extent and shape of the orange part of elytra and in the shape of the posterior margin of phallobase (Figs 54–55).

###### Description.

Body medium-sized, dark brown to black, only pronotum testaceous to brown and basal quarter of elytra orange (Fig. 9). Head small, including eyes slightly narrower than frontal margin of pronotum, antennal tubercles small. Eyes hemispherically prominent, eye diameter 2.3 times frontal interocular distance. Antennae compressed, pubescent, length of antennomere 3 1.1 times antennomere 2. Pronotum transverse, 1.7 times wider than long at midline, anterior margin widely rounded, semicircular, without prominent anterior angles, posterior margin of pronotum bisinuate, surface of disc glabrous, with sparse long pubescence (Fig. 24). Elytra almost parallel-sided, 3.0 times longer than width at humeri; slightly widened posteriorly, widest at apical fourth. Elytral costae inconspicuous. Legs compressed, with dense pubescence. Male genitalia with short rounded parameres bearing slender membranous processes; phallus curved, phallobase wide, deeply emarginate (Figs 54–55).

###### Measurements.

BL 6.0 mm, PL 0.8 mm, PW 1.3 mm, HW 1.7 mm, Edist 0.79 mm, Ediam 0.35 mm.

###### Distribution.

Indonesia: Sumatra.

###### Etymology.

The specific epithet refers to the type locality of the holotype.

##### Platerodrilus sibayakensis sp. n.

Taxon classificationAnimaliaColeopteraLycidae

http://zoobank.org/3A4F0BBB-BA23-498D-8C1E-E39CA46F1D84

Figs 10 , 25 , 71–72

###### Material examined.

Holotype. Male (LMBC, UPOL 001389), Sumatra Utara, Brastagi, Gn. Sibayak, 26. Jan.–1. Feb. 2005, 1600–2200 m. Paratypes. 2 males (LBMC), Sumatra, SW of Brastagi, Gn. Sinabung, 22. Feb. 1991, 1300–1800 m. Paratype. Male (LBMC), Sumatra, SW of Brastagi, Gn. Sinabung, 19.–23. Feb. 1991, 1300–1800 m. Paratype. Male (LBMC, UPOL 001372), Sumatra, Utara, Brastagi, Gn. Sibayak, 26. Jan.–1. Feb. 2005, 1600–2200 m. Paratype. Male (LBMC), Sumatra, Sinabung, Mar. 1998.

###### Diagnosis.

*Platerodrilus sibayakensis* and *Platerodrilus angustatus* were recovered as sister species (Fig. 1) and they share the pronotum with acutely projected posterior angles. *Platerodrilus sibayakensis* differs in the 4.1 times longer antennomere 4 than its width in the middle and the more robust phallus tapering gradually from the base to apex (Figs 71–72).

###### Description.

Body medium-sized, dark brown to black, pronotum testaceous to brown and basal third of elytra orange (Fig. 10). Head small, including eyes slightly narrower than frontal margin of pronotum, antennal tubercles small. Eyes hemispherically prominent, eye diameter 1.7 times frontal interocular distance. Antennae compressed, pubescent, length of antennomere 3 0.7 times antennomere 2. Pronotum transverse, 1.9 times wider than long at midline, anterior margin almost straight, with marked anterior angles, lateral margins straight, posterior margin of pronotum bisinuate, posterior angles acutely projected, surface of disc weakly glabrous, finely punctuate, with long pubescence (Fig. 25). Elytra almost parallel-sided, elytra 3.5 times longer than width at humeri; slightly widened posteriorly, widest at apical fourth, elytral costae inconspicuous. Legs compressed, with dense pubescence. Male genitalia with short rounded parameres bearing slender membranous processes; phallus curved, phallobase wide, deeply emarginate (Figs 71–72).

###### Measurements.

BL 8.5 mm, PL 0.9 mm, PW 1.8 mm, HW 2.0 mm, Edist 0.73 mm, Ediam 0.44 mm.

###### Distribution.

Indonesia: Sumatra.

###### Etymology.

The specific epithet refers to the type locality of the holotype.

##### Platerodrilus sinabungensis sp. n.

Taxon classificationAnimaliaColeopteraLycidae

http://zoobank.org/4275B855-5901-45B6-BD6A-CF6EB0BFB430

Figs 11 , 26 , 58–59

###### Material examined.

Holotype. Male (LMBC), Sumatra, SW of Brastagi, Gn. Sinabung, 22. Feb. 1991, 1300–1500 m. Paratypes. 2 males (LBMC), Sumatra, SW of Brastagi, Gn. Sinabung, 22. Feb. 1991, 1400–1900 m.

###### Diagnosis.

*Platerodrilus sinabungensis* resembles *Platerodrilus montanus* in the dark coloured pronotum. These species differ in the shape of the phallobase (Figs 58–59, 66).

###### Description.

Body medium-sized, dark brown to black, only basal two fifths of elytra orange testaceous (Fig. 11). Head including eyes slightly wider than frontal margin of pronotum. Eye hemispherically prominent, their diameter 2.3 times frontal interocular distance. Antennae compressed, antennomere 3 as long as antennomere 2. Head and antennae with short dense pubescence. Pronotum transverse, 1.7 wider than long at midline, anterior and lateral margins weakly rounded, posterior margin of pronotum simply rounded to straight in middle, disc bare in middle, pubescent along lateral margins (Fig. 26). Elytra with inconspicuous carinae. Elytra 3.4 times longer than width at humeri, elytra widest posteriorly. Legs compressed with dense pubescence. Male genitalia with curved phallus and short rounded parameres bearing slender membranous processes with basal part serrate; phallobase wide, widely emarginate (Figs 58–59).

###### Measurements.

BL 8.9 mm, PL 1.0 mm, PW 1.7 mm, HW 2.1 mm, Edist 0.89 mm, Ediam 0.4 mm.

###### Distribution.

Indonesia: Sumatra.

###### Etymology.

The specific epithet refers to the type locality of the holotype.

##### Platerodrilus talamauensis sp. n.

Taxon classificationAnimaliaColeopteraLycidae

http://zoobank.org/FB6FE156-5B2D-4232-897F-62EC10EFFB1F

Figs 15 , 29 , 52–53

###### Material examined.

Holotype. Male (LMBC, UPOL 001376), Sumatra Barat, Pasaman, Gn. Talamau, 14.–15. Jan. 2005, 1000 m. Paratype. Male (LMBC, UPOL 001375), Sumatra Barat, Pasaman, Gn. Talamau, 14.–15. Jan. 2005, 1000 m.

###### Diagnosis.

*Platerodrilus talamauensis* and *Platerodrilus ranauensis* are closely related (Fig. 1) and they differ in the extent and shape of the orange part of the elytra (Figs 9, 15) and in the shape of posterior margin of phallobase (Figs 52–55).

###### Description.

Male. Body medium-sized, dark brown to black, only pronotum, mesoscutellum and basal three fifths of elytra orange (Fig. 15). Head small, with eyes slightly wider than frontal margin of pronotum. Eyes hemispherically prominent, eye diameter 2.4 times frontal interocular distance. Antennae compressed, reaching two thirds of elytral length, antennomere 3 0.9 times antennomere 2. Head and antennae densely pubescent. Pronotum transverse, 1.2 wider than long at midline. Anterior margin of pronotum rounded, anterior angles inconspicuous, posterior margin bisinuate (Fig. 29). Elytra with inconspicuous carinae, parallel-sided, 2.9 times longer than width at humeri, widest posteriorly. Legs compressed with dense pubescence. Male genitalia with curved phallus, phallus twice longer than apical processes of parameres, phallobase widely emarginate. (Fig. 52–53).

###### Measurements.

BL 6.5 mm, PL 1.0 mm, PW 1.6 mm, HW 1.9 mm, Edist 0.88 mm, Ediam 0.36 mm.

###### Distribution.

Indonesia: Sumatra.

###### Etymology.

The specific epithet refers to the type locality of the holotype.

##### Platerodrilus tujuhensis sp. n.

Taxon classificationAnimaliaColeopteraLycidae

http://zoobank.org/52952DAE-6457-4DF0-B57C-E4A7F0989063

Figs 12 , 27 , 41 , 56–57

###### Material examined.

Holotype. Male (LMBC, UPOL 001385), Sumatra, Jambi Kersik Tua, Gn. Kerinci, 19–22. Jan. 2005, 1600–2200 m. Paratype. Female (LMBC, VP2305), Sumatra, Jambi prov. Kerinci Seblat N. P., 7 km E Kayuaro, Mt. Tujuh, 1°45'S, 101°25'E, 25. Feb.–2. Mar. 2003, 1750 ± 250 m.

###### Diagnosis.

*Platerodrilus tujuhensis* resembles in the uniformly light coloured head, pronotum and elytra *Platerodrilus robinsoni*, which differs in the more robust basal part of the phallus. The molecular phylogeny suggests the sister relationships of *Platerodrilus maninjauensis* and *Platerodrilus tujuhensis*. These species differ in colouration (Figs 7, 12) and the shape of the phallus and phallobase (Figs 56–57, 60–61). The female larvae of both species are similar and differ only in the relative size of mesonotal tubercles, which are smaller in *Platerodrilus tujuhensis*.

###### Description.

Body dark brown to black; head, pronotum and elytra yellow to orange, apical margins of elytra infuscate (Fig. 12). Head including eyes narrower than pronotum, antennal tubercles slightly prominent. Eyes hemispherically prominent, frontal interocular distance 2.6 times eye diameter. Antennae compressed, slender, length of antennomere 3 0.9 times antennomere 2. Pronotum transverse, 1.8 wider than long at midline, anterior margin widely rounded, anterior angles inconspicuous, posterior margin bisinuate (Fig. 27). Elytra 3.7 times longer than width at humeri, widest posteriorly, elytral costae inconspicuous. Legs slender, compressed with dense pubescence. Male genitalia with slender, curved phallus and small rounded parameres bearing slender membranous processes, phallobase wide, narrowly and deeply emarginate (Figs 56–57).

Female larva. Body flat, wide, dark brown, only margins of tergites lighter, pronotum triangular (Fig. 41), with two glabrous rounded tubercles postero-laterally, another two tubercles in middle of anterior margin; mesothorax strongly transverse, with rounded lateral margins and weakly projected posterior angles, laterally with four tubercles, upper rounded, lower transverse, metathorax similar in shape with more acutely projected posterior margins. Abdominal segments with slender and long lateral processes.

###### Measurements.

BL 8.1 mm, PL 0.9 mm, PW 1.6 mm, HW 1.9 mm, Edist 0.81 mm, Ediam 0.31 mm. Larva. BL 32.7 mm, PL 6.6 mm, PW 10.2 mm.

###### Distribution.

Indonesia: Sumatra.

###### Etymology.

The specific epithet refers to the locality of the paratype.

##### Platerodrilus robinsoni

Taxon classificationAnimaliaColeopteraLycidae

Blair, 1928

Figs 6 , 19 , 62–63

Platerodrilus korinchiana robinsoni Blair 1928: 182.
Platerodrilus robinsoni Blair, 1928, stat. n.

###### Material examined.

Holotype. Sumatra, Sungei Kumbang, Korinchi, 4500 ft, Apr. 1914 (BMNH). Other material examined. Male (LMBC, UPOL 001378), Sumatra, Jambi Kersik Tua, Gn. Kerinci, 19.–22. Jan. 2005, 1600–2200 m.

###### Diagnosis.

*Platerodrilus robinsoni* resembles *Platerodrilus luteus* in general appearance, but differs in the shape of the phallobase and phallus (Figs 62–63, 67–68).

###### Redescription.

Body black; head and mesoscutellum dark brown, pronotum and elytra orange yellow; antennae, and legs dark brown to black (Fig. 6). Head small, antennal tubercles separated by deep groove. Eyes hemispherically prominent, frontal interocular distance 2.1 times maximum eye diameter. Antennae slender, compressed, densely pubescent, antennomere 3 1.1 times longer than antennomere 2. Pronotum transverse, 1.6 times wider than long, anterior margin rounded, frontal angles obtuse, lateral margins slightly convex, posterior angles approximately rectangular (Fig. 19). Elytra parallel-sided, elytra 3.3 times longer than width at humeri, elytral costae weak, covered with dense pubescence. Male genitalia with curved phallus; parameres short, rounded, with slender membranous process; phallobase wide, deeply emarginate (Figs 62–63).

###### Measurements.

BL 7.8 mm, PL 1.0 mm, PW 1.6 mm, HW 2.0 mm, Edist 0.85 mm, Ediam 0.40 mm.

###### Distribution.

Indonesia: Sumatra.

###### Remark.

*Platerodrilus robinsoni* differs from *Platerodrilus korinchianus* in the colouration; but both species are syntopic. Therefore, the species rank is proposed for *Platerodrilus robinsoni* stat. n.

##### Platerodrilus ruficollis

Taxon classificationAnimaliaColeopteraLycidae

Pic, 1942 comb. n.

Fig. 39

Falsocalochromus ruficollis Pic, 1942
Duliticola hoiseni Wong, 1996: 175, syn. n.

###### Material examined.

Holotype, male, Presqu'ile Malaise (MNHP).

###### Remark.

Wong (1996) described and illustrated *Duliticola hoiseni*. During the recent search in the Paris Museum we found that Pic (1942) described the same species as *Falsocalochromus ruficollis* and placed in the relationships with *Calochromus* despite that fact that the species perfectly fits in his own concept of *Platerodrilus*. Therefore, we propose *Duliticola hoiseni* as a junior subjective synonym of *Platerodrilus ruficollis*.

#### Species incertae sedis

##### Platerodrilus testaceicollis

Taxon classificationAnimaliaColeopteraLycidae

Pic, 1921: 14.

###### Remark.

*Platerodrilus testaceicollis* was placed in *Platerodrilus* by Kazantsev, 2009, but the abdomen of the type is missing and the species cannot be placed in any species group unless further specimen is available. The redescription was given by Wong (1998).

##### Duliticola javanica

Taxon classificationAnimaliaColeopteraLycidae

Kemner, 1928: 136.

###### Remark.

The type series contains just female larvae judging from the illustrations might include two species. The adult is unknown.

## Supplementary Material

XML Treatment for Platerodrilus

XML Treatment for Platerodrilus paradoxus

XML Treatment for Platerodrilus foliaceus

XML Treatment for Platerodrilus wongi

XML Treatment for Platerodrilus ngi

XML Treatment for Platerodrilus wittmeri

XML Treatment for Platerodrilus ijenensis

XML Treatment for Platerodrilus korinchianus

XML Treatment for Platerodrilus luteus

XML Treatment for Platerodrilus maninjauensis

XML Treatment for Platerodrilus montanus

XML Treatment for Platerodrilus palawanensis

XML Treatment for Platerodrilus ranauensis

XML Treatment for Platerodrilus sibayakensis

XML Treatment for Platerodrilus sinabungensis

XML Treatment for Platerodrilus talamauensis

XML Treatment for Platerodrilus tujuhensis

XML Treatment for Platerodrilus robinsoni

XML Treatment for Platerodrilus ruficollis

XML Treatment for Platerodrilus testaceicollis

XML Treatment for Duliticola javanica
